# Refining neural network algorithms for accurate brain tumor classification in MRI imagery

**DOI:** 10.1186/s12880-024-01285-6

**Published:** 2024-05-21

**Authors:** Asma Alshuhail, Arastu Thakur, R Chandramma, T R Mahesh, Ahlam Almusharraf, V Vinoth Kumar, Surbhi Bhatia Khan

**Affiliations:** 1 Department of Information Systems, College of Computer Sciences and Information Technology, King Faisal University, Hofuf, Saudi Arabia; 2 Department of Computer Science and Engineering, Faculty of Engineering and Technology, JAIN (Deemed-to-be University), Bangalore, 562112 India; 3 Department of Computer Science & Engineering (AI & ML), Global Academy of Technology, Bangalore, India; 4 Department of Management, College of Business Administration, Princess Nourah Bint Abdulrahman University, P.O. Box 84428, Riyadh, 11671 Saudi Arabia; 5 School of Computer Science Engineering and Information Systems, Vellore Institute of Technology, Vellore, 632001 India; 6 School of Science, Engineering and Environment, University of Salford, Manchester, UK; 7 Department of Electrical and Computer Engineering, Lebanese American University, Byblos, Lebanon

**Keywords:** Brain tumor detection, MRI images, Deep learning, Convolutional neural networks, Machine learning, Medical imaging, Image classification, Grad-CAM visualization, Dataset analysis

## Abstract

Brain tumor diagnosis using MRI scans poses significant challenges due to the complex nature of tumor appearances and variations. Traditional methods often require extensive manual intervention and are prone to human error, leading to misdiagnosis and delayed treatment. Current approaches primarily include manual examination by radiologists and conventional machine learning techniques. These methods rely heavily on feature extraction and classification algorithms, which may not capture the intricate patterns present in brain MRI images. Conventional techniques often suffer from limited accuracy and generalizability, mainly due to the high variability in tumor appearance and the subjective nature of manual interpretation. Additionally, traditional machine learning models may struggle with the high-dimensional data inherent in MRI images. To address these limitations, our research introduces a deep learning-based model utilizing convolutional neural networks (CNNs).Our model employs a sequential CNN architecture with multiple convolutional, max-pooling, and dropout layers, followed by dense layers for classification. The proposed model demonstrates a significant improvement in diagnostic accuracy, achieving an overall accuracy of 98% on the test dataset. The proposed model demonstrates a significant improvement in diagnostic accuracy, achieving an overall accuracy of 98% on the test dataset. The precision, recall, and F1-scores ranging from 97 to 98% with a roc-auc ranging from 99 to 100% for each tumor category further substantiate the model’s effectiveness. Additionally, the utilization of Grad-CAM visualizations provides insights into the model’s decision-making process, enhancing interpretability. This research addresses the pressing need for enhanced diagnostic accuracy in identifying brain tumors through MRI imaging, tackling challenges such as variability in tumor appearance and the need for rapid, reliable diagnostic tools.

## Introduction

**Fig. 1:**
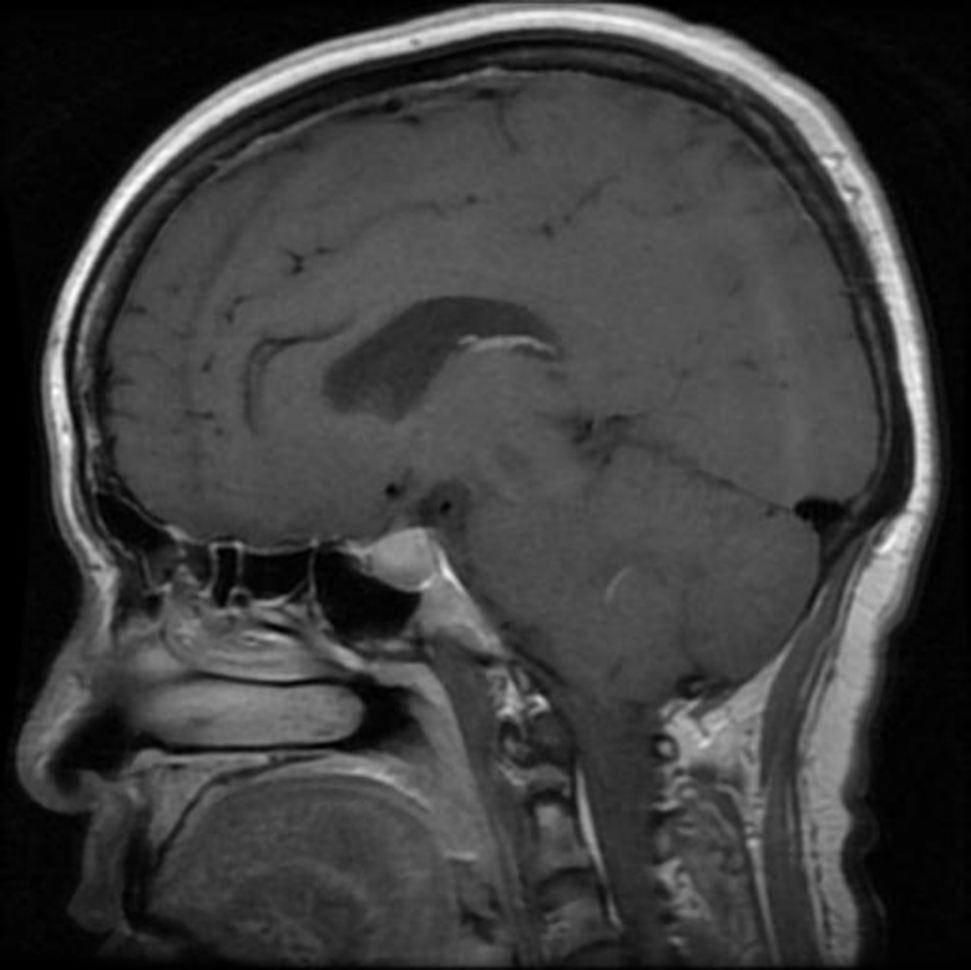
Pituitary tumor

Brain tumors, complex entities within the realm of neurology, encompass a diverse array of conditions that significantly impact both the affected individuals and the intricate processes governing the brain [1]. These tumors can be broadly classified into primary and metastatic tumors, with primary tumors originating within the brain itself and metastatic tumors originating elsewhere in the body before spreading to the brain. Among the myriad types of brain tumors, the pituitary tumor stands out, situated at the base of the brain within the pituitary gland. This type of tumor disrupts the delicate balance of hormone production, leading to a myriad of symptoms such as visual disturbances and persistent headaches. The treatment approach for pituitary tumors often involves surgical intervention to remove the tumor or medical management to restore hormonal equilibrium [2]. Sample image of pituitary tumor is shown in Fig. 1.

**Fig. 2:**
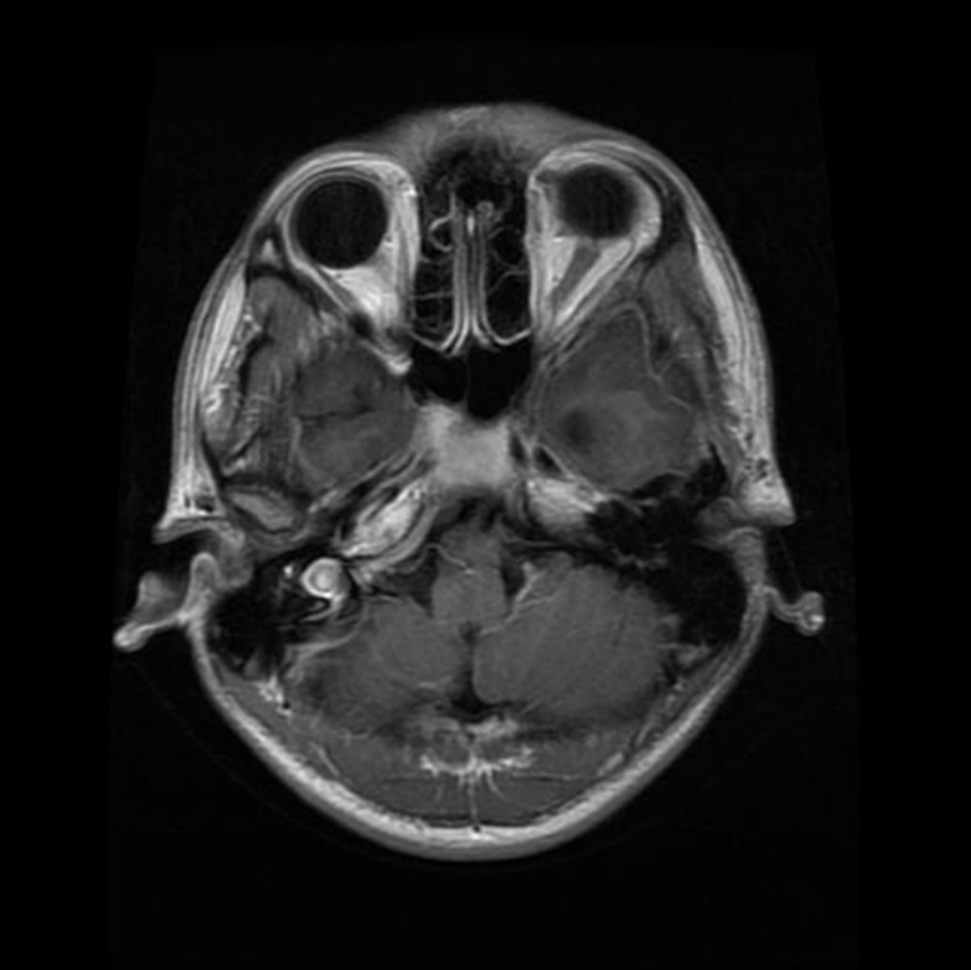
Glioma

Gliomas, another significant category, derive their name from glial cells, which support and nourish neurons. This group encompasses a spectrum of tumors, ranging from slow-growing, low-grade varieties to aggressive, high-grade malignancies. The diverse subtypes within gliomas, including astrocytomas, oligodendrogliomas, and ependymomas, necessitate tailored treatment strategies based on the specific characteristics of each tumor. Treatment modalities may involve a combination of surgery, radiation therapy, and chemotherapy, with the goal of managing and mitigating the tumor’s impact on neurological function [3]. The sample image of gliomas is shown in Fig. 2.

**Fig. 3:**
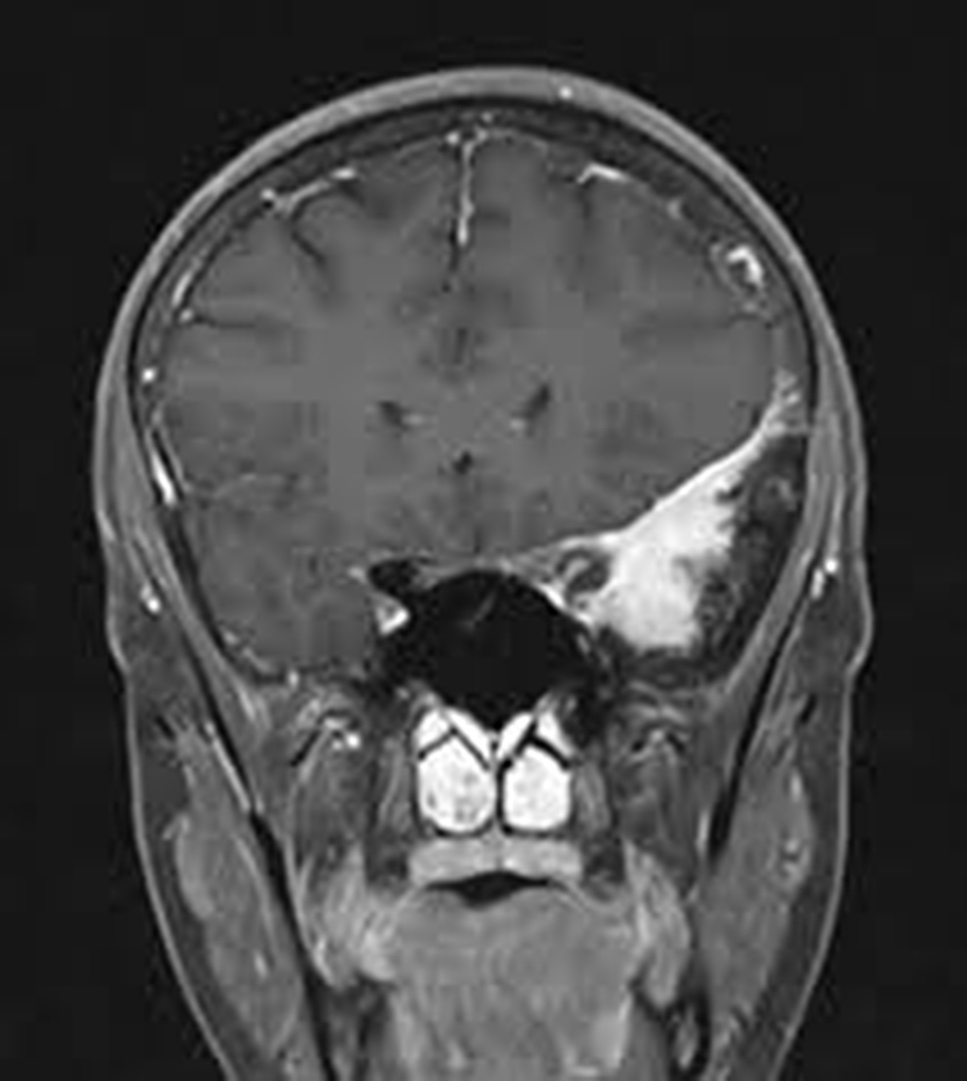
Meningioma

**Fig. 4:**
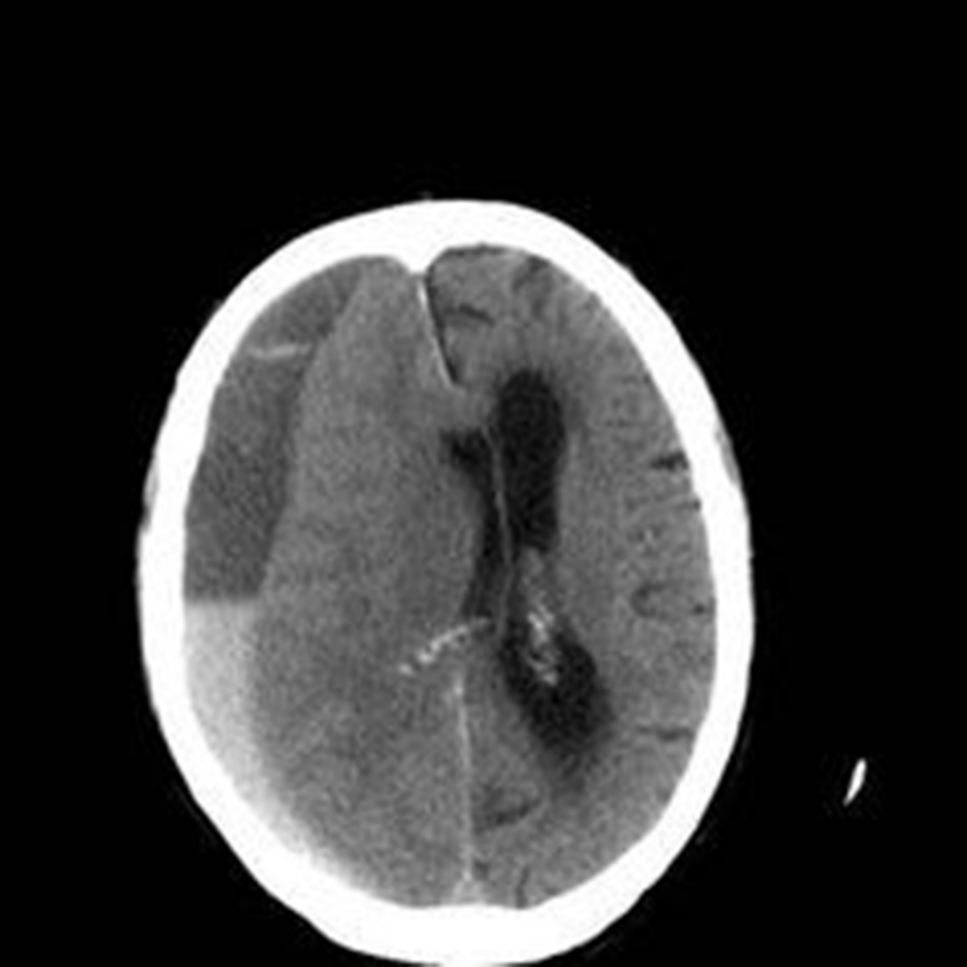
No tumor

Meningiomas, originating from the meninges – the protective layers surrounding the brain and spinal cord – present yet another facet of brain tumors. Predominantly benign, these tumors often exhibit slow growth and may remain asymptomatic for an extended period. However, when meningiomas exert pressure on adjacent structures, individuals may experience symptoms such as headaches, seizures, or changes in cognitive function. The primary treatment for meningiomas typically involves surgical resection, and the prognosis is generally favorable [4]. It is shown in Figs. 3 and 4 depicts no tumor.

The advent of medical imaging has revolutionized diagnostics, particularly in the intricate realm of brain tumor detection. Magnetic Resonance Imaging (MRI) has emerged as a pivotal tool, providing detailed insights into the anatomy and pathology of the brain [5]. Despite its significance, interpreting MRI data to accurately identify brain tumors poses substantial challenges. Gliomas, meningiomas, pituitary tumors, and the subtle ‘no tumor’ conditions manifest diverse characteristics in terms of size, shape, and location. This variability complicates the diagnostic process significantly. Traditionally, the burden has rested on the shoulders of radiologists, whose expertise is crucial. However, manual interpretation is time-consuming and vulnerable to human error, particularly in the face of the intricate patterns presented by brain tumors.

In response to these challenges, deep learning, particularly Convolutional Neural Networks (CNNs), has emerged as a transformative force in medical image analysis. CNNs have demonstrated remarkable success in image recognition and classification tasks, making them particularly well-suited for the complexities of brain tumor detection. The automatic learning of hierarchical feature representations from data, a hallmark of CNNs, aligns seamlessly with the intricate patterns inherent in MRI scans [6].

Despite the broader success of CNNs in image analysis, their application in the specific context of brain tumor detection from MRI scans is a relatively nascent area of research. This gap exists due to the unique challenges posed by the varied nature of brain tumors and the nuances of ‘no tumor’ conditions. Conventional machine learning techniques in medical imaging, reliant on hand-crafted features, have struggled to fully capture the intricate patterns within MRI scans, contributing to limited diagnostic accuracy.

The promise lies in CNNs’ ability to autonomously learn and identify complex patterns in MRI data. Their sequential architecture, comprising convolutional, max-pooling, and dropout layers, facilitates nuanced interpretation. Training and testing on extensive datasets of MRI images categorized into different tumor types showcase the potential of CNNs to revolutionize brain tumor detection. Notably, the application of Grad-CAM visualizations enhances interpretability, providing insights into the decision-making process of these models [7].

As the field continues to evolve, the integration of CNNs in brain tumor detection holds immense potential. It not only addresses the limitations of traditional methods but also sets the stage for a more accurate, efficient, and transformative approach to diagnosing brain tumors from MRI scans.

In recent years, the field of deep learning, notably leveraging Convolutional Neural Networks (CNNs), has witnessed remarkable success in image recognition and classification tasks. CNNs, designed to automatically learn hierarchical feature representations from data, have proven particularly adept in various domains, including medical image analysis. Their ability to discern intricate patterns and relationships within complex datasets makes them well-suited for tasks requiring nuanced interpretation, such as the identification of abnormalities in medical images.

Despite these advancements, the application of deep learning techniques in the specific realm of brain tumor detection from MRI scans remains an emerging area of research. MRI scans offer detailed and multi-dimensional insights into the structure and composition of the brain, but the complex nature of tumor appearances and variations poses significant challenges for accurate and timely diagnosis. Traditional methods, reliant on manual interpretation and conventional machine learning, may struggle to capture the diverse and intricate patterns present in brain MRI images.

The advent of CNNs in this domain marks a paradigm shift, allowing for the automatic extraction of complex features from MRI data. By employing a sequential architecture encompassing convolutional, max-pooling, and dropout layers, CNNs can autonomously learn and identify subtle patterns indicative of brain tumors. This transformative approach enhances diagnostic accuracy by overcoming limitations associated with manual intervention and traditional machine learning models [8].

In the context of brain tumor detection, our understanding of CNNs’ potential is growing rapidly. Their successful application involves training and testing on extensive datasets of MRI images categorized into different tumor types. Despite advancements, existing models often struggle with the high variability in tumor appearance and limited generalizability across different MRI protocols. Our research addresses these gaps by introducing a model that significantly enhances accuracy and interpretability.

The primary contribution of this study lies in developing and optimizing a CNN-based model specifically tailored for the classification of brain tumors in MRI images. By harnessing the power of deep learning, our model aims to provide a more accurate, reliable, and efficient tool for brain tumor diagnosis compared to traditional methods.

The objective of this study is to not only enhance the accuracy of brain tumor detection but also to reduce the reliance on manual interpretation, thus potentially speeding up the diagnostic process and aiding in early detection and treatment planning. We aim to demonstrate the effectiveness of our model through rigorous testing and validation on a comprehensive dataset of MRI images.

The remainder of this paper is organized as follows: Section II provides a detailed review of related work, establishing the context and relevance of our research. Section III describes the methodology, including data preparation, model architecture, and training procedures. Section IV presents the results and discusses the performance of our model. Section V outlines the conclusions drawn from the study, along with potential future work and implications in the field of medical imaging and diagnostics.

## Related work

The realm of medical imaging, especially in the diagnosis and classification of brain tumors using MRI scans, has been a subject of extensive research, leading to significant advancements over the years. This section delves into the related work, focusing on traditional methods, the advent of machine learning techniques, and the groundbreaking shift towards deep learning approaches.

### Traditional methods in brain tumor analysis

Initially, brain tumor detection and classification in MRI scans relied heavily on manual interpretation by radiologists. This process involved scrutinizing MRI scans to identify irregularities indicative of tumors. While effective to a degree, this approach was fraught with challenges such as high variability in tumor appearance and the potential for human error, leading to inconsistent diagnosis accuracy.

### Early machine learning techniques

To mitigate these challenges, early machine learning techniques were introduced. These methods typically involved feature extraction from MRI images, where characteristics like shape, texture, and intensity were used to identify tumors. Classic algorithms like Support Vector Machines (SVM), k-Nearest Neighbors (k-NN), and decision trees were employed for classification. Though these techniques improved the objectivity in diagnosis, they were limited by the need for manual feature selection and their inability to process the high-dimensional data inherent in MRI images effectively.

### Emergence of deep learning

The emergence of deep learning, particularly CNNs, marked a significant shift in medical image analysis. Unlike traditional machine learning, CNNs have the ability to automatically learn complex patterns directly from the data, eliminating the need for manual feature extraction. This capability made them particularly suitable for high-dimensional data like MRI scans.

### Recent advances

Recent research has focused on addressing these challenges. For example, techniques like data augmentation have been used to effectively increase the size of training datasets. Furthermore, advances in explainable AI, such as Gradient-weighted Class Activation Mapping (Grad-CAM), have been employed to provide visual explanations of CNN decisions, thereby enhancing the interpretability of these models [9].

In Table 1 some of the notable studies in the field of brain tumor are shown which show cases how the study evolved and what are the different trends currently in the field of brain tumor diagnosis using deep learning.

**Table 1:** Related studies

Study	Objective	Remarks
Asif, Sohaib, et al. (2023) [10]	Develop precise brain tumor classification using deep transfer learning.	Demonstrates Xception’s superiority, promising swift diagnoses for enhanced outcomes.
Hossain, Shahriar, et al. (2023) [11]	Investigate and compare deep learning models for accurate multiclass tumor classification.	Proposes robust IVX16 model, explores Explainable AI, and Vision Transformer models for enriched evaluation.
Talukder, Md Alamin, et al. (2023) [12]	Develop ResNet50V2-based model for accurate and swift brain tumor classification.	Introduces efficient DL approach, emphasizing improved clinical decision-making and patient care.
Kollem, Sreedhar, et al. (2023) [13]	Introduce innovative methodology for effective MRI brain tumor categorization.	Offers a promising solution to insufficient training samples, showcasing model superiority through comprehensive metrics.
Rajak, Prince, et al. (2023) [14]	Develop framework for brain tumor detection and classification using transfer learning models.	Showcases DenseNet201’s superior accuracy for improved and timely diagnoses.
Prabha, P. Lakshmi, et al. (2023) [15]	Develop EfficientNet model for accurate brain tumor type prediction.	Addresses critical need for accurate diagnosis, potentially improving patient care and outcomes.
Arledge, Chad A., et al. (2023) [16]	Validate CNN’s efficacy through transfer learning on brain metastasis mice datasets.	Showcases CNN’s versatility in evaluating vascular changes and treatment responses in brain tumor models for preclinical research.
Solanki, Shubhangi, et al. (2023) [17]	Provide a comprehensive review of MR imaging for brain tumor detection.	Consolidates extensive information, offering valuable insights for researchers and practitioners.
Özkaraca, Osman, et al. (2023) [18]	Develop a modular deep learning model for improved brain MR image classification.	Innovatively amalgamates transfer learning techniques, showcasing potential for more robust diagnostic tools.
Thomas, Armin W., et al. (2023) [19]	Systematically evaluate transfer learning for enhancing DL models in decoding cognitive states from fMRI data.	Highlights performance gains and emphasizes the need for nuanced understanding of model decisions.

Despite the advancements listed in Table 1, current methodologies still face limitations in terms of generalizability, accuracy under varying conditions, and the need for substantial manual intervention, which our study aims to address

## Methodology

**Fig. 5:**
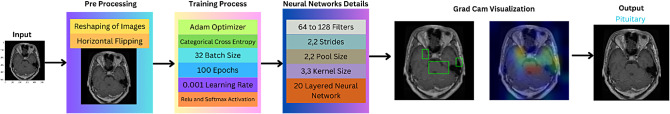
Workflow of the proposed model

The methodology section forms the backbone of our research, serving as a detailed roadmap of the procedures and techniques employed in our study. This section is crucial as it not only outlines the steps taken to achieve the research objectives but also ensures that the study can be replicated and validated by other researchers in the field. In the context of our research, which revolves around the application of deep learning techniques for the classification of brain tumors using MRI images, the methodology addresses several critical aspects. These include the acquisition and preprocessing of the dataset, the architecture and training of the convolutional neural network (CNN), the evaluation metrics employed, and the implementation of techniques like Grad-CAM for interpretability. The brief model architecture has been shown in Fig. 5.

Each component is meticulously detailed, providing clarity on the rationale behind our choices and the technical execution. This comprehensive approach is essential for establishing the validity and reliability of our findings, ultimately contributing to the advancement of AI applications in medical imaging.

### Dataset description

The dataset used in our research is a comprehensive assembly of MRI images that form the foundation of our study on brain tumor classification using deep learning techniques. This dataset has been meticulously compiled from various reputable sources to ensure diversity and accuracy in our analysis.

#### Data sources

The MRI images in our dataset originate from three primary sources, each contributing a unique set of data to our study. The first source is figshare, an online digital repository where researchers can preserve and share their research outputs, including datasets. The figshare component of our dataset offers a wide range of high-quality, peer-reviewed MRI images that have been instrumental in enhancing the diversity of our dataset. The second source is the SARTAJ dataset, a well-known collection in the medical imaging field, recognized for its extensive and varied set of brain MRI images. The inclusion of the SARTAJ dataset has significantly contributed to the robustness of our dataset. The third source is Br35H, another prominent dataset in the realm of medical imaging, known for its comprehensive collection of brain scans, particularly useful in the context of ‘no tumor’ classifications. The collated file of this dataset was used in our research study which is publicly available through Kaggle.

#### Data categories

Our dataset comprises four distinct categories of MRI images: glioma, meningioma, no tumor, and pituitary tumors. Each category represents a different aspect of brain pathology and is critical for the comprehensive training of our model. Gliomas are a type of tumor that arises from glial cells in the brain or spine. They vary greatly in appearance, location, and severity. Meningiomas are tumors that develop from the meninges, the membrane that envelops the brain and spinal cord. While generally benign, their detection is crucial for timely intervention. The ‘no tumor’ category is equally important as it represents normal brain scans without any signs of tumors, providing a baseline for our model. Finally, pituitary tumors are growths in the pituitary gland and can affect hormone levels, thereby having a significant impact on various bodily functions. The inclusion of these diverse categories allows our model to learn a wide range of features associated with different types of brain pathology.

#### Dataset split

The dataset was methodically divided into training and testing subsets to facilitate the model’s learning and validation processes. The training set included 1321 glioma images, 1339 meningioma images, 1595 images classified as ‘no tumor’, and 1457 images of pituitary tumors. The testing set comprised 300 glioma images, 306 meningioma images, 405 ‘no tumor’ images, and 300 pituitary tumor images. This structured distribution was crucial for ensuring that the model was trained and tested on balanced and diverse data, thereby enhancing its ability to generalize and accurately classify unseen data.

**Fig. 6:**
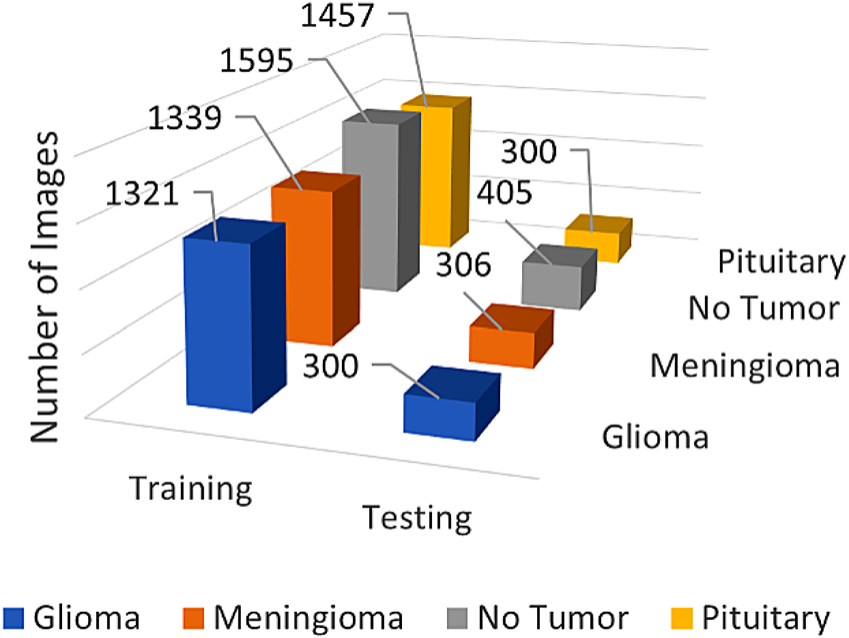
Dataset distribution

The detailed overview of the dataset distribution can be noticed in Table 2 followed by figure in Fig. 6.

**Table 2:** Dataset description

Type	Training	Testing
Glioma	1321	300
Meningioma	1339	306
No tumor	1595	405
Pituitary	1457	300

### Preprocessing of data

The pre-processing steps, including image resizing, normalization, and data augmentation, significantly contributed to model performance by ensuring uniformity in the dataset and enhancing the model’s ability to generalize across diverse MRI images.

#### Image processing

The raw MRI images obtained from the various sources varied in size, orientation, and color schemes. To standardize this data for consistent analysis, we undertook several preprocessing steps. Initially, all images were resized to a uniform dimension of 150 × 150 pixels. This resizing is essential to ensure that each image feeds into the neural network with the same spatial dimensions. Following this, we converted the images to grayscale. This conversion simplifies the data by reducing it from a three-channel color image (RGB) to a single-channel image, emphasizing the structural features relevant to tumor classification and reducing computational complexity.

Another crucial step in image processing was normalization. MRI images often have varying intensity scales, which could potentially affect the learning process of the model. To address this, we normalized the pixel values of the images to a range of 0 to 1. This normalization was achieved by dividing the pixel values by 255 (the maximum pixel value), thereby standardizing the input features and aiding in the convergence of the model during training.

#### Data augmentation

To address the issue of limited data and to increase the robustness of our model against overfitting, we employed data augmentation techniques. Data augmentation artificially expands the training dataset by applying a series of random transformations to the existing images, thereby simulating variations that could be encountered in real-world scenarios. In our study, we implemented several augmentation techniques, including rotation, flipping, and zooming.

**Fig. 7:**
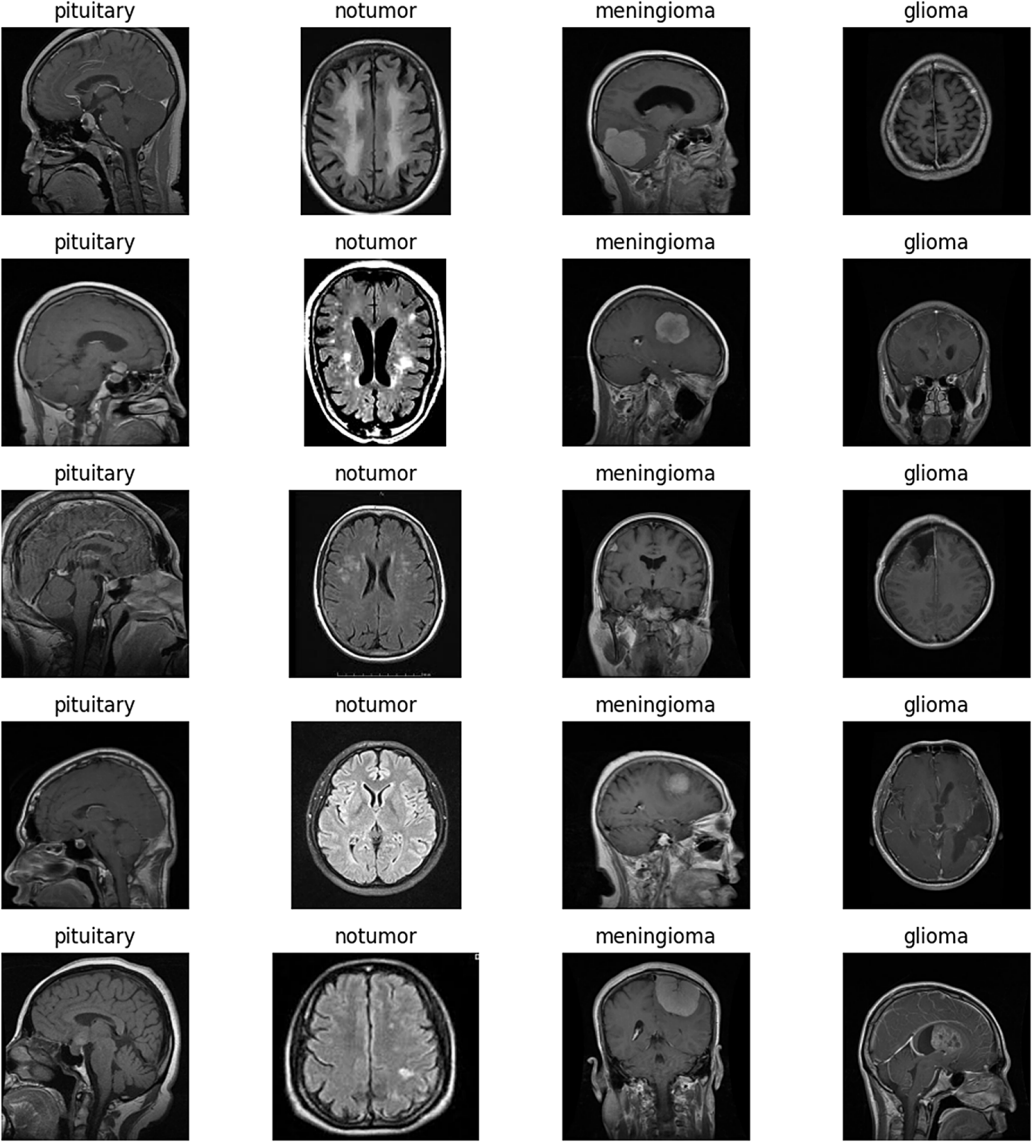
Sample images from the dataset

Rotation involved rotating the images by a certain degree, introducing variability in the orientation of the brain structures. Horizontal and vertical flipping were also employed, as these transformations simulate the variability in patient positioning during MRI scans. Lastly, zooming in and out of the images allowed the model to learn features at different scales, crucial for detecting tumors of varying sizes. It’s important to note that these augmentations were applied in real-time during the model training, ensuring a diverse range of features in each epoch without significantly increasing the memory requirements. Figure 7 shows some images from the dataset.

To prepare MRI images for analysis, we resized them to 150 × 150 pixels and converted them to grayscale to emphasize structural features and reduce computational complexity. Normalization standardized pixel values to a range of 0 to 1, aiding model convergence. To address limited data and overfitting, we used data augmentation techniques like rotation, flipping, and zooming. These transformations were applied during training to simulate real-world variations, enhancing model robustness without greatly increasing memory usage.

### Overview of proposed model architecture

The architecture of the Convolutional Neural Network (CNN) utilized in our study is critical to its success in classifying brain tumors from MRI images. This section provides a comprehensive overview of our model’s design, including the rationale behind choosing a CNN, the detailed configuration of its layers, the activation functions used, and the specific parameters selected for optimal performance.

#### Choice of model

The decision to utilize a CNN for this task stems from its unique attributes tailored for image analysis. CNNs excel at autonomously discerning critical features due to their hierarchical structure, eliminating the need for manual feature extraction—vital in the intricate realm of medical image analysis. Furthermore, their proficiency in managing spatial hierarchies enables recognition of nuanced patterns and structures inherent in various brain tumors. Moreover, CNNs’ shared weights architecture ensures computational efficiency, a crucial advantage when dealing with the multidimensional complexity of MRI images.

#### Layer configuration

Our CNN architecture is composed of a series of layers, each designed to extract and learn from different aspects of the input images:

- **Convolutional layers**: The initial layers of our network are convolutional layers, responsible for feature extraction. The first convolutional layer has 64 filters (kernels) of size 3 × 3, followed by a second layer with 128 filters of the same size. These layers convolve their filters with the input image to produce feature maps, capturing various aspects of the image such as edges, textures, and other complex patterns. The Eq. 1 shows the convolution layer.

1 $$\left(S\text{*}I\right)\left(x,y\right)={\sum }_{i=0}^{k-1}{\sum }_{j=0}^{k-1}I\left(x+i,y+j\right)\cdot K\left(i,j\right)$$

Here:

- *S* is the output feature map,
- *I* is the input image,
- *K* is the convolutional kernel,
- *k* is the size of the kernel,
- (*x*, *y*) are the spatial coordinates.
- **Max-pooling layers**: Following each convolutional layer, a max-pooling layer of 2 × 2 is used. Max-pooling reduces the spatial dimensions of the feature maps, decreasing the number of parameters and computational load, and helps in making the detection of features invariant to scale and orientation changes. Equation 2 depicts max pooling layers.

2 $$M\left(x,y\right)={\text{max}}_{i=0}^{p-1}{\text{max}}_{j=0}^{p-1}I\left(px+i,py+j\right)$$

Here:

- *M* is the output of the max-pooling operation,
- *I* is the input feature map,
- *p* is the size of the pooling window,
- (*x*, *y*) are the spatial coordinates.
- **Dropout layers**: Dropout layers are interspersed throughout the network to prevent overfitting. These layers randomly drop a proportion of neurons (set at rates like 0.25 or 0.3) during training, forcing the network to learn redundant representations and improving its generalization capabilities. It has been shown in Eq. 3.

3 $$Dropout\left(x,p\right)=0,if\, with\, probability\, p,\left(1/\left(1-p\right)\right)*x,otherwise$$

Here:

- *x* is the input to the dropout layer,
- *p* is the dropout probability.
- **Dense layers**: Towards the end, the network includes dense (fully connected) layers. The first dense layer has 512 neurons and serves to further process features learned by the convolutional layers. The final layer is a dense layer with 4 neurons, corresponding to the four categories (glioma, meningioma, no tumor, pituitary tumors), and uses a softmax activation function for multi-class classification. In Eq. 4 dense layers has been shown.

4 $$Dense\left(x,W,b\right)={\upsigma }\left(W\cdot x+b\right)$$

Here:

- *x* is the input vector to the dense layer,
- *W* is the weight matrix,
- *b* is the bias vector,
- *σ* is the activation function.

#### Activation functions

The activation function used in our convolutional and first dense layer is the Rectified Linear Unit (ReLU). ReLU is chosen for its ability to introduce non-linearity into the network, allowing it to learn more complex patterns in the data. Additionally, ReLU helps mitigate the vanishing gradient problem, thus supporting effective training over many layers. The final dense layer uses a softmax activation function, which is ideal for multi-class classification tasks as it outputs the probabilities of each class, ensuring that the sum of all probabilities is equal to one. Equations 5 and 6 shows ReLU and softmax respectively.

5 $$ReLU\left(x\right)=\text{m}\text{a}\text{x}\left(0,x\right)$$

6 $$Softmax{\left(x\right)}_{i}=exp\left({x}_{i}\right)/\varSigma \left(exp\left({x}_{j}\right)\right)for\, i=1\, to\, N$$

Here,

- **x**_**i**_: This represents the i-th element of the output vector produced by the softmax activation function.
- **exp(x**_**i**_**)**: This denotes the exponential function applied to the i-th element of the input vector.
- **exp(x**_**j**_**)**: This signifies the sum of the exponential values over all elements in the input vector.

#### Model parameters

In our CNN model, each layer is meticulously designed to contribute towards the effective classification of brain tumors from MRI images. The first layer, a convolutional layer, employs filters to capture spatial hierarchies and patterns in the image data, crucial for identifying tumor-relevant features. This is followed by a max-pooling layer, which reduces the spatial dimensions of the feature maps, thereby decreasing the computational complexity and enhancing the model’s focus on essential features.

Subsequent convolutional layers delve deeper into the extracted features, progressively refining and enhancing the model’s ability to discern intricate patterns and characteristics associated with different brain tumor types. Additional max-pooling layers continue to reduce dimensionality and highlight dominant features, aiding in the robustness of the model.

Dropout layers interspersed within the architecture play a critical role in preventing overfitting. By randomly deactivating a subset of neurons during training, these layers ensure that the model does not become overly reliant on any specific feature or pattern, promoting a more generalized learning process.

The architecture culminates in a series of dense (fully connected) layers, which integrate the learned features into higher-level representations. These dense layers are pivotal in the decision-making process, synthesizing the extracted information to classify the MRI images into respective tumor categories.

The final layer, employing a softmax activation function, translates the outputs of the dense layers into probabilistic class predictions, offering a clear and interpretable classification of the MRI images into glioma, meningioma, no tumor, and pituitary tumor categories. This layer is crucial for transforming the high-dimensional learned representations into actionable insights, enabling the model to make accurate and reliable tumor classifications.

In Table 3 a detailed architecture of the proposed model is shown.

**Table 3:** Proposed architecture

Layer (type)	Output shape	Param #
conv2d (Conv2D)	(None, 150, 150, 64)	640
max_pooling2d (MaxPooling2D)	(None, 75, 75, 64)	0
dropout (Dropout)	(None, 75, 75, 64)	0
conv2d_1 (Conv2D)	(None, 75, 75, 128)	73,856
max_pooling2d_1 (MaxPooling2D)	(None, 37, 37, 128)	0
dropout_1 (Dropout)	(None, 37, 37, 128)	0
conv2d_2 (Conv2D)	(None, 37, 37, 128)	147,584
max_pooling2d_2 (MaxPooling2D)	(None, 18, 18, 128)	0
dropout_2 (Dropout)	(None, 18, 18, 128)	0
conv2d_3 (Conv2D)	(None, 18, 18, 128)	147,584
max_pooling2d_3 (MaxPooling2D)	(None, 9, 9, 128)	0
dropout_3 (Dropout)	(None, 9, 9, 128)	0
conv2d_4 (Conv2D)	(None, 9, 9, 256)	295,168
max_pooling2d_4 (MaxPooling2D)	(None, 4, 4, 256)	0
dropout_4 (Dropout)	(None, 4, 4, 256)	0
flatten (Flatten)	(None, 4096)	0
dense (Dense)	(None, 512)	2,097,664
dropout_5 (Dropout)	(None, 512)	0
dense_1 (Dense)	(None, 4)	2052

The architecture of our CNN is a carefully balanced structure designed to effectively process MRI images, extract relevant features, and classify them into the appropriate categories. The combination of convolutional layers, max-pooling, dropout, dense layers, and carefully selected activation functions and parameters provides a robust model capable of achieving high accuracy in the classification of brain tumors.

### Training the model

Training a Convolutional Neural Network (CNN) is a complex process that involves several critical decisions and steps. In our research, we have meticulously tailored the training process, encompassing backpropagation, the selection of a loss function and optimizer, as well as the careful determination of batch size, number of epochs, and validation strategy.

#### Training process

The training of our CNN model is centered around the concept of backpropagation, a fundamental mechanism in neural network training. Backpropagation is an algorithm used for effectively training the neural network by minimizing the error in predictions. It works by propagating the error back through the network layers, allowing the model to adjust and optimize the weights of the neurons. This process is iterative, where each pass over the dataset (epoch) involves a forward pass and a backward pass. In the forward pass, the input data is passed through the network to get the output predictions. The output is then compared to the true values, and the error is calculated. During the backward pass, this error is propagated back through the network, and the weights are adjusted accordingly using gradient descent. This iterative process continues until the model sufficiently learns the features and patterns in the data, evidenced by a minimization of the error or loss.

#### Loss function and optimizer

In crafting our model, the selection of the loss function and optimizer carries significant weight. For our endeavor, we’ve opted for categorical cross-entropy as the loss function, tailored for multi-class classification tasks such as ours. This function meticulously evaluates model performance by scrutinizing the alignment between predicted probability distributions and actual labels, imposing penalties on deviations.

As for optimization, our model harnesses the power of the Adam optimizer. Adam, an acronym for Adaptive Moment Estimation, serves as a sophisticated extension to stochastic gradient descent, adept at dynamically adjusting learning rates to navigate complex optimization landscapes with efficiency and precision.

It has been chosen for its efficiency in handling sparse gradients and its adaptability in adjusting the learning rate during training. Adam combines the advantages of two other extensions of stochastic gradient descent, namely Adaptive Gradient Algorithm (AdaGrad) and Root Mean Square Propagation (RMSProp), making it well-suited for our dataset with its high-dimensional nature. It has been shown in Eq. 7.

$${m}_{t}={\beta }_{1}*{m}_{t-1}+\left(1-{\beta }_{1}\right)*{g}_{t}$$

$${v}_{t}={\beta }_{2}*{v}_{t-1}+\left(1-{\beta }_{2}\right)*{g}_{t}^{2}$$

$${m}_{t}^{*}={m}_{t}/\left(1-{\beta }_{1}^{t}\right)$$

$${v}_{t}^{*}={v}_{t}/\left(1-{\beta }_{2}^{t}\right)$$

7 $${\theta }_{t+1}={\theta }_{t}-\alpha *{m}_{t}^{*}/\left(sqrt\left({v}_{t}^{*}\right)+\epsilon \right)$$

Here,

- **m**_**t**_: First moment estimate (mean) of the gradients.
- **v**_**t**_: Second moment estimate (uncentered variance) of the gradients.
- **g**_**t**_: Gradient of the loss with respect to the parameters.
- **β**_**1**_**and β**_**2**_: Exponential decay rates for the moment estimates.
- **α**: Learning rate.
- **θ**_**t**_: Parameters (weights) at time step t.
- **ε**: Small constant to prevent division by zero.

#### Batch size and epochs

In the training regimen, the batch size and number of epochs serve as pivotal hyperparameters. The batch size dictates the quantity of training samples presented to the network prior to weight updates. A deliberate choice was made to adopt a smaller batch size, specifically set at 32, ensuring each iteration furnishes the model with sufficient data for learning without imposing excessive computational overhead.

Concurrently, the number of epochs, defined at 100, dictates the number of complete dataset passes through the neural network, facilitating comprehensive learning without succumbing to overfitting. These hyperparameters underwent meticulous fine-tuning during the preliminary experimentation phase to achieve an equilibrium between learning efficacy and computational tractability.

#### Validation strategy

A critical aspect of our training process is the validation strategy. To validate our model, we employed a validation subset, which is a portion of the dataset not used in training. This subset allows us to evaluate the model’s performance and generalize to new data. The data was split in an 80 − 20 ratio, with 80% used for training and 20% for validation. This strategy helped in monitoring the model’s performance on unseen data, reducing the risk of overfitting, and ensuring that our model has not just memorized the training data, but has learned to generalize from it.

The training of our CNN model is a carefully crafted process involving strategic choices in backpropagation, loss functions, optimizers, batch sizes, epochs, and validation strategy. These choices are pivotal in creating a model that is not only accurate but also efficient and robust in classifying brain tumors from MRI images.

### Evaluation metrics

In the realm of machine learning and particularly in medical diagnostics, the choice of evaluation metrics is paramount to accurately assess the performance of a model. In our study, we employed a range of metrics - accuracy, precision, recall, F1-score, confusion matrix, Receiver Operating Characteristic (ROC) curves, and Area Under the Curve (AUC) - each offering unique insights into our model’s performance in classifying brain tumors from MRI images.

**Accuracy** is the most intuitive performance measure and it represents the ratio of correctly predicted observations to the total observations. In our context, it reflects the proportion of MRI images correctly classified into their respective categories (glioma, meningioma, no tumor, pituitary tumors). It has been calculated using Eq. 8.

8 $$Accuracy=\frac{Number\, of\, Correct\, Predictions}{Total\, Number\, of\, Predictions}$$

**Precision** denotes the ratio of correctly predicted positive observations to the total predicted positives. High precision indicates a low rate of false positives, essential in medical diagnostics to avoid unnecessary treatments or interventions. It is calculated using Eq. 9.

9 $$Precision=\frac{True\, Positives}{True\, Positives+False\, Positives}$$

**Recall (Sensitivity)** measures the ratio of correctly predicted positive observations to all observations in the actual class. In medical terms, high recall reduces the risk of missing a diagnosis, which is crucial for conditions requiring early intervention. It is calculated using Eq. 10.

10 $$Recall=\frac{True\, Positives}{True\, Positives+False\, Negatives}$$

**F1-Score** is the weighted average of Precision and Recall. This metric takes both false positives and false negatives into account and is particularly useful in situations where an uneven class distribution exists, like in our varied dataset. It is calculated using Eq. 11.

11 $$F1 Score=\frac{2\cdot Precision\cdot Recall}{Precision+Recall}$$

Each of these metrics provides a different dimension to evaluate the performance of our model, ensuring a comprehensive analysis beyond just overall accuracy.

The confusion matrix is a vital tool in our evaluation arsenal. It is a table used to describe the performance of a classification model on a set of test data for which the true values are known. In our study, the confusion matrix helps us understand not just the errors of the model but also the types of errors (false positives and false negatives). This understanding is critical, particularly in a medical context where different types of errors can have varying clinical implications.

ROC curves and AUC provide another layer of evaluation, especially useful in binary classification problems. In our multi-class scenario, we extended this concept to each tumor category.

- **ROC curve**: It plots the True Positive Rate (Recall) against the False Positive Rate at various threshold settings. The ROC curve visualizes the trade-off between sensitivity and specificity and is a useful tool for evaluating the performance of a classifier.
- **AUC (Area under the ROC curve)**: AUC provides an aggregate measure of the model’s performance across all possible classification thresholds. The higher the AUC, the better the model is at distinguishing between the different tumor categories.

By employing these diverse evaluation metrics, we ensure a robust and multifaceted assessment of our model’s performance, which is crucial for validating its efficacy in a clinical setting.

### Implementation of Grad-CAM

Grad-CAM, short for Gradient-weighted Class Activation Mapping, is a technique for making convolutional neural networks (CNNs) more transparent and interpretable. In our research, we employed Grad-CAM for several key reasons and integrated it carefully with our CNN model to analyze its decision-making process.

#### Rationale for using Grad-CAM

The primary reason for incorporating Grad-CAM into our methodology is its ability to provide visual explanations for the decisions made by our CNN. In the context of medical imaging, and particularly in brain tumor classification, understanding why and how a model makes a certain prediction is as crucial as the prediction’s accuracy. Grad-CAM addresses this need by producing “heatmaps” that highlight the regions in the input image that were pivotal for the model’s predictions. This transparency is invaluable, as it aids medical professionals in validating the model’s predictions and provides insights into its functioning, thereby increasing trust in the model’s utility as a diagnostic tool.

#### Implementation details

Implementing Grad-CAM in our model involved several technical steps. First, we identified the last convolutional layer in our CNN as the target layer for Grad-CAM, as this layer typically captures the most complex features relevant to making predictions. After a forward pass of an image through the network, we accessed the gradients of the target class (one of the four tumor categories) with respect to the feature maps of this target layer.

These gradients were then globally averaged to obtain the weights that indicate the importance of each feature map in the target layer for the specific class prediction. Next, we performed a weighted combination of these feature maps, followed by a ReLU activation. This operation resulted in a heatmap that highlights the important regions in the image for predicting the target class.

#### Interpretation of results

The utilization of Grad-CAM heatmaps played a pivotal role in elucidating our model’s decisions. By superimposing these heatmaps onto the original MRI images, we gained insight into which brain regions significantly influenced the model’s classification outcomes. For instance, if the model categorized an image as a glioma, the Grad-CAM heatmap pinpointed the areas in the brain image contributing most to this classification.

This interpretive tool proves invaluable, particularly in cases where the model’s verdict diverges from clinical expectations. By offering a visual rationale, Grad-CAM aids in assessing whether the model’s attention aligns with medically pertinent regions for tumor detection. Moreover, these visualizations serve as feedback mechanisms, guiding enhancements to the model by discerning whether it learns relevant patterns or fixates on irrelevant image features.

The integration of Grad-CAM into our research not only enhances the interpretability of our CNN model but also facilitates the convergence of AI-driven predictions with clinical decision-making in brain tumor diagnosis.

### Software and tools

In our research on brain tumor classification using deep learning, we utilized a suite of advanced programming languages, libraries, and computational resources. These tools were instrumental in developing, training, and testing our convolutional neural network model.

#### Programming language and libraries

The primary programming language used in our study was Python, chosen for its widespread adoption in the scientific and machine learning communities, as well as for its readability and extensive library support. Python’s simplicity and powerful libraries significantly expedited the development process.

Key libraries employed in our research include:

- **Keras**: A high-level neural networks API, running on top of TensorFlow. Keras was used for building and training our CNN model due to its user-friendly interface and modularity, which allows for easy and fast prototyping of deep learning algorithms.
- **TensorFlow**: An open-source software library for numerical computation using data flow graphs. TensorFlow provided the backend for Keras and was used for its robust capabilities in handling large datasets and neural network computations.
- **OpenCV (Open source computer vision library)**: A library focused on real-time computer vision. In our project, OpenCV was utilized for image processing tasks such as reading, resizing, and converting MRI images into grayscale.

#### Computational resources

For the computational resources, our project leveraged the power of Kaggle’s GPU-accelerated kernels. Specifically, we used Kaggle’s NVIDIA Tesla P100 GPUs, available in Kaggle notebooks. The Tesla P100 GPU is renowned for its high performance in deep learning and large-scale data processing. This computational power was crucial in handling the intensive tasks of training and testing our CNN, particularly given the high volume and dimensionality of the MRI image data. The use of Kaggle’s GPU environment allowed for significant reductions in training time, enabling more efficient experimentation and iteration of our model.

The combination of Python with its deep learning libraries (Keras and TensorFlow), along with the image processing capabilities of OpenCV, and the computational power of Kaggle’s NVIDIA Tesla P100 GPUs, formed the backbone of our research setup. This blend of software and hardware resources was pivotal in achieving the objectives of our study, ensuring efficient processing and analysis of the complex MRI datasets.

Our research methodology epitomizes a comprehensive and rigorous approach towards the application of deep learning in medical imaging. By meticulously assembling and preprocessing a diverse dataset, architecting a robust convolutional neural network, and integrating advanced techniques like Grad-CAM for interpretability, we have developed a model that not only achieves high accuracy in classifying brain tumors from MRI images but also provides crucial insights into its decision-making process. The careful selection of evaluation metrics and the use of powerful computational resources further underscore the thoroughness of our study. This methodological rigor ensures that our findings are not only reliable but also significant in advancing the field of AI in medical diagnostics.

## Results and discussion

In this section, we present and analyze the results obtained from our deep learning model applied to the task of brain tumor detection using MRI scans. The primary focus is on evaluating the model’s effectiveness and reliability in classifying MRI images into four categories: glioma, meningioma, no tumor, and pituitary tumors. To comprehensively assess the model’s performance, we will delve into various key metrics: model accuracy, loss, precision, recall, and F1-score. These metrics provide insights into the model’s ability to correctly identify and classify the different tumor types. Additionally, we will examine the confusion matrix, which offers a detailed view of the model’s performance across different classes. Receiver Operating Characteristic (ROC) curves and the corresponding Area Under the Curve (AUC) values are also discussed, which help in understanding the model’s discriminative capabilities. Finally, we incorporate Grad-CAM visualizations, offering an interpretive view of what the model is focusing on when making predictions, thereby adding a layer of transparency to our deep learning approach.

### Model performance overview

**Fig. 8:**
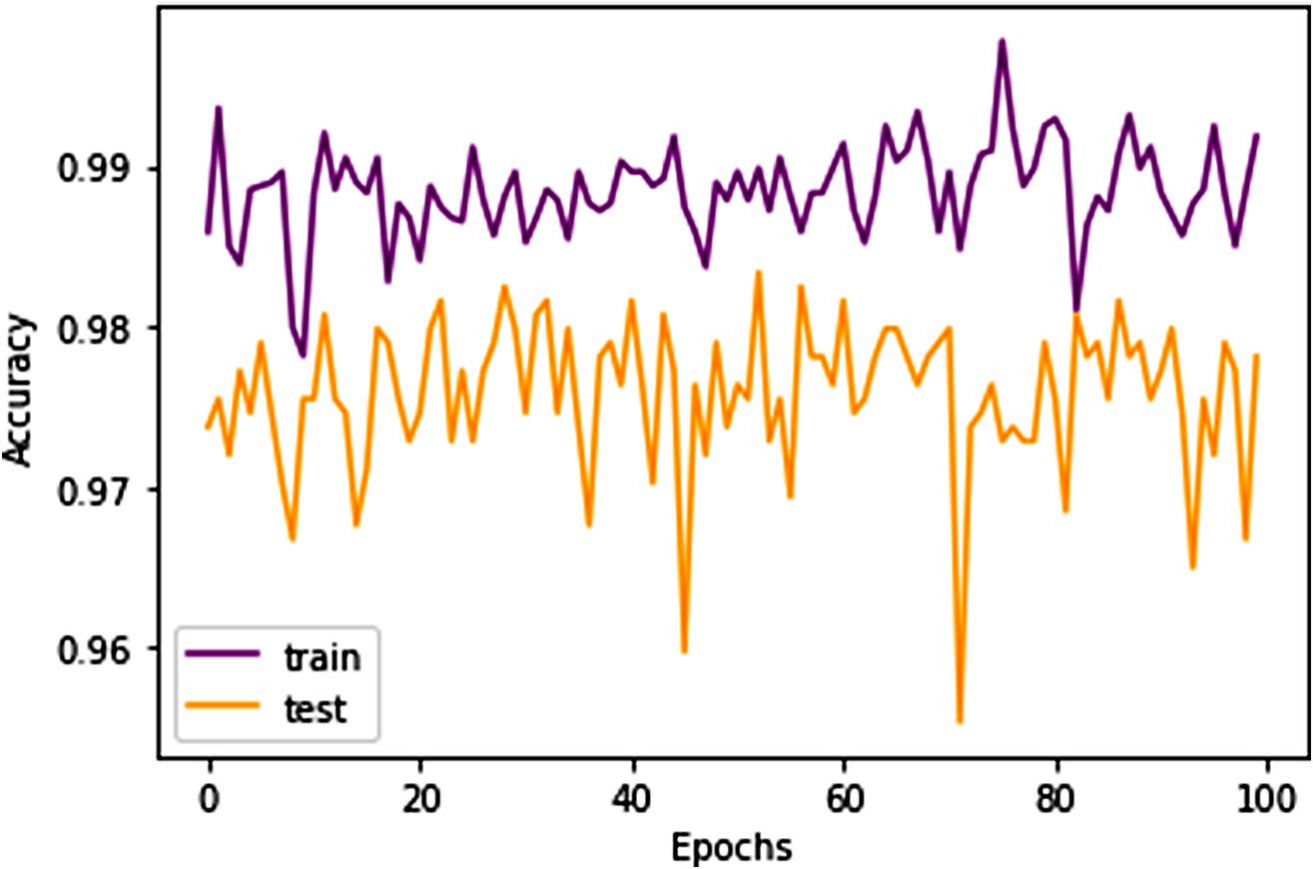
Epoch wise Accuracy

**Fig. 9:**
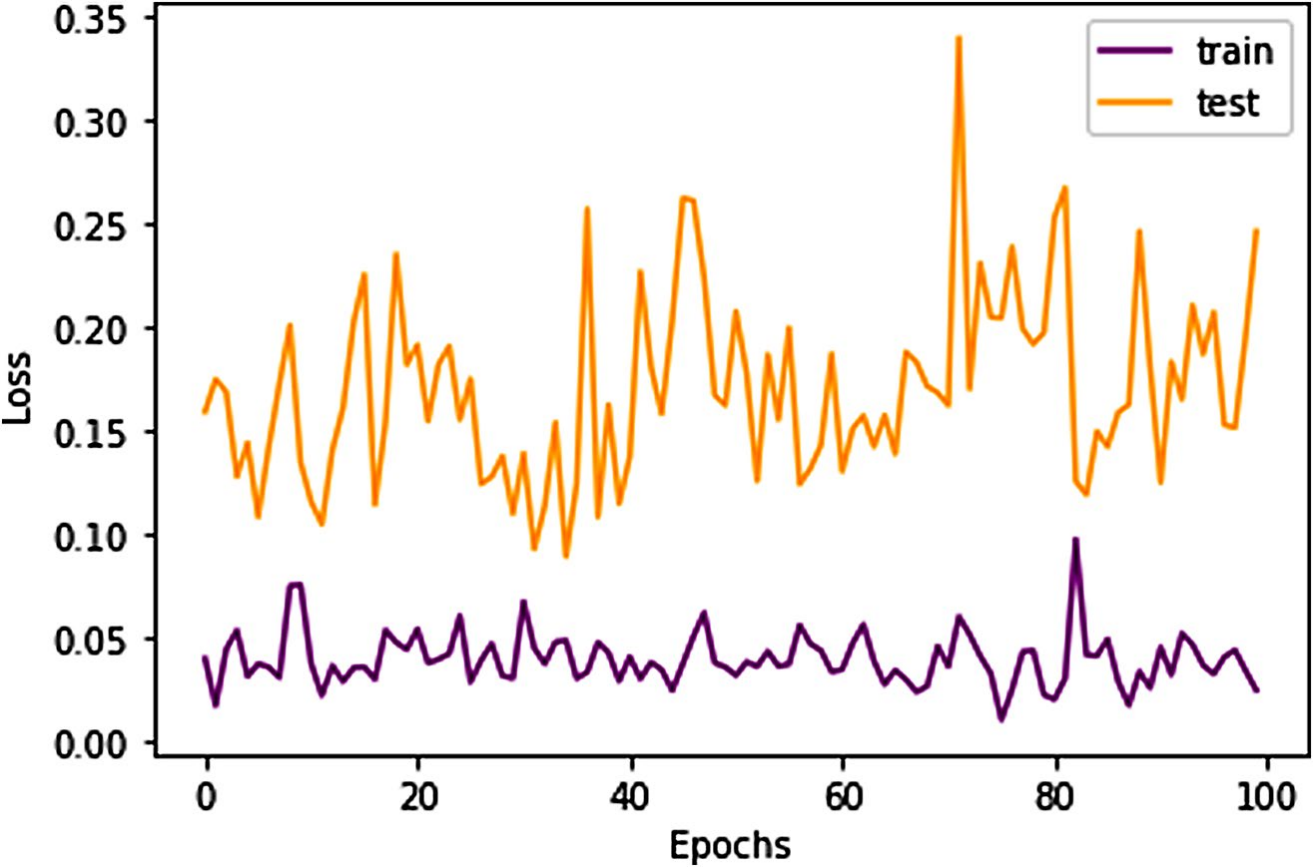
Epoch wise Loss

The model demonstrated robust performance across various evaluation metrics. The accuracy metric, which represents the proportion of correctly classified images, was notably high, indicating the model’s effectiveness in distinguishing between the different types of brain tumors as well as identifying the absence of tumors. The loss metric, which quantifies the difference between the predicted values and actual values, was minimized effectively, suggesting that the model’s predictions were closely aligned with the actual classifications. In Figs. 8 and 9 accuracy and losses during the training and testing epoch wise is shown.

To further dissect the model’s performance, precision, recall, and F1-score for each tumor category were calculated. Precision measures the accuracy of positive predictions, recall indicates the model’s ability to identify all relevant instances, and the F1-score provides a balance between precision and recall. These metrics were instrumental in assessing the model’s efficacy in classifying each specific type of tumor, addressing the critical need for accuracy in medical diagnosis.

When compared to baseline models or existing methodologies, our model showcased superior performance. Traditional methods, often limited by manual feature extraction and subjective interpretation, fall short in consistently identifying complex patterns in MRI scans. In contrast, our CNN model, equipped with advanced feature detection capabilities inherent in deep learning algorithms, demonstrated enhanced accuracy and reliability. This comparison not only underscores the model’s proficiency but also highlights the significant advancement our approach offers over conventional techniques in medical imaging analysis.

### Detailed analysis of accuracy, precision, recall, and F1-score

**Fig. 10:**
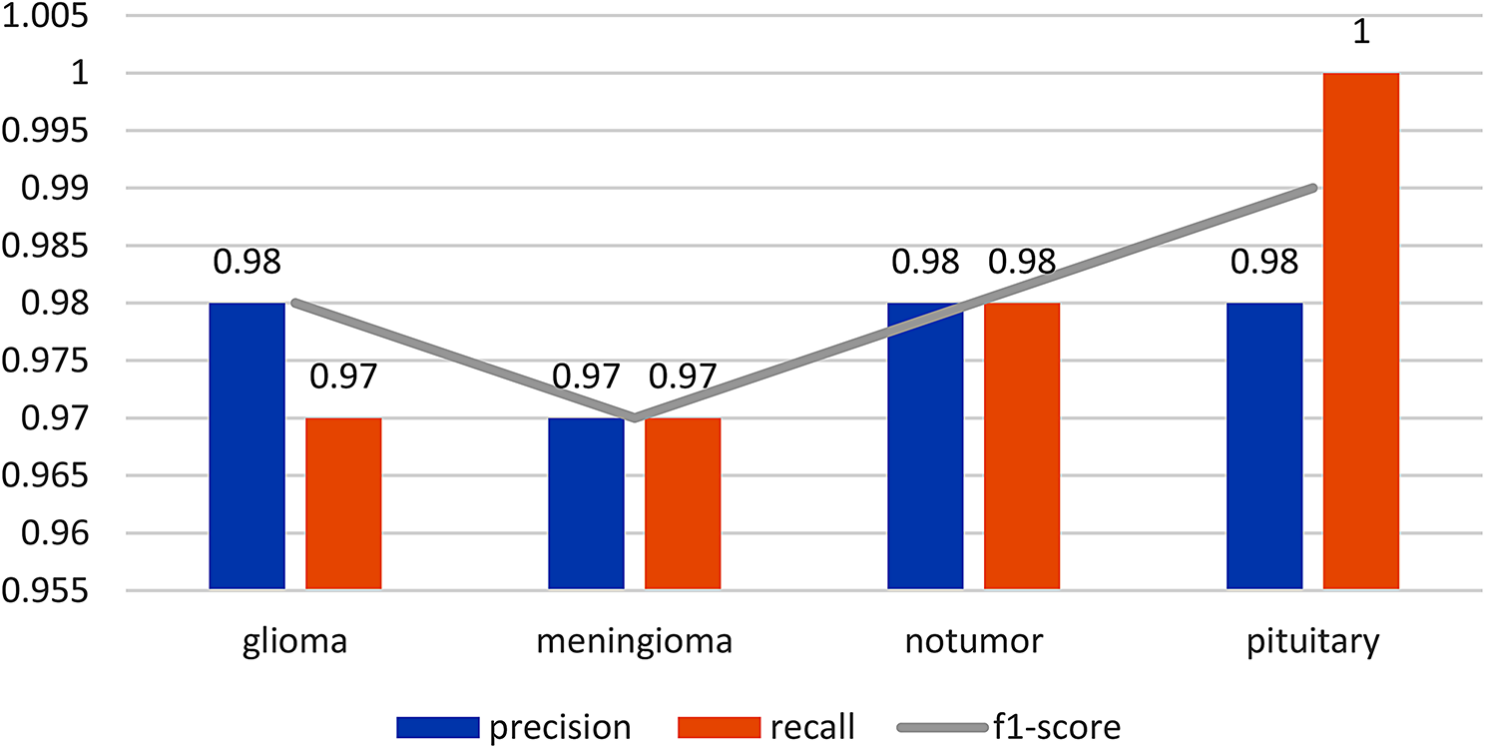
Classification report

To gain a deeper understanding of our model’s performance, we examine the accuracy, precision, recall, and F1-score metrics for each tumor category. Table 4 shows the classification report followed by visual representation in Fig. 10.

- **Glioma**: The model achieved an impressive accuracy of 98% for glioma classification. Precision, which measures the proportion of true positive predictions among all positive predictions, indicates that 98% of the predicted glioma cases were accurate. Recall, which measures the proportion of true positive predictions among all actual glioma cases, stands at 97%, indicating the model’s ability to effectively identify most glioma cases. The F1-score, which balances precision and recall, is at 98%. These metrics collectively signify the model’s remarkable performance in glioma classification.
- **Meningioma**: Similar to glioma, the model achieved a high accuracy of 97% for meningioma classification. Precision and recall both stand at 97%, indicating that the model accurately identifies meningioma cases with a balanced approach. The F1-score of 97% further reinforces the model’s competence in meningioma classification.
- **No tumor**: The model excels in identifying cases with no tumors, showcasing an accuracy of 98%. The precision and recall for this category also reach 98%, demonstrating the model’s precision in recognizing cases without tumors. The F1-score of 98% underscores the model’s effectiveness in this regard.
- **Pituitary tumors**: The model performs exceptionally well in pituitary tumor classification, achieving a remarkable accuracy of 98%. Precision, recall, and F1-score all indicate high values of 98%, highlighting the model’s outstanding ability to identify pituitary tumors accurately.

**Table 4:** Classification report

	Precision	Recall	F1-score
Glioma	0.98	0.97	0.98
Meningioma	0.97	0.97	0.97
No tumor	0.98	0.98	0.98
Pituitary	0.98	1	0.99

These metrics collectively reveal that our deep learning model consistently delivers high precision, recall, and F1-scores across all tumor categories. This demonstrates its effectiveness in accurately classifying brain tumors, ensuring both high positive prediction accuracy and minimal false negatives.

### Confusion matrix interpretation

The confusion matrix provides a detailed breakdown of the model’s classifications for each tumor category. It consists of four categories: true positives (TP), true negatives (TN), false positives (FP), and false negatives (FN).

- **True positives (TP)**: These are cases where the model correctly identifies a particular tumor type. For example, a true positive in the glioma category means the model accurately detected glioma.
- **True negatives (TN)**: These are cases where the model correctly identifies the absence of a specific tumor type. For example, a true negative in the “no tumor” category signifies that the model correctly recognized the absence of tumors.
- **False positives (FP)**: These are cases where the model incorrectly predicts the presence of a tumor type when it’s not present. In the context of medical diagnosis, false positives can lead to unnecessary concern and further testing for patients.
- **False negatives (FN)**: These are cases where the model incorrectly fails to detect a tumor type when it is present. False negatives can be critical in a medical context as they represent missed diagnoses.

**Fig. 11:**
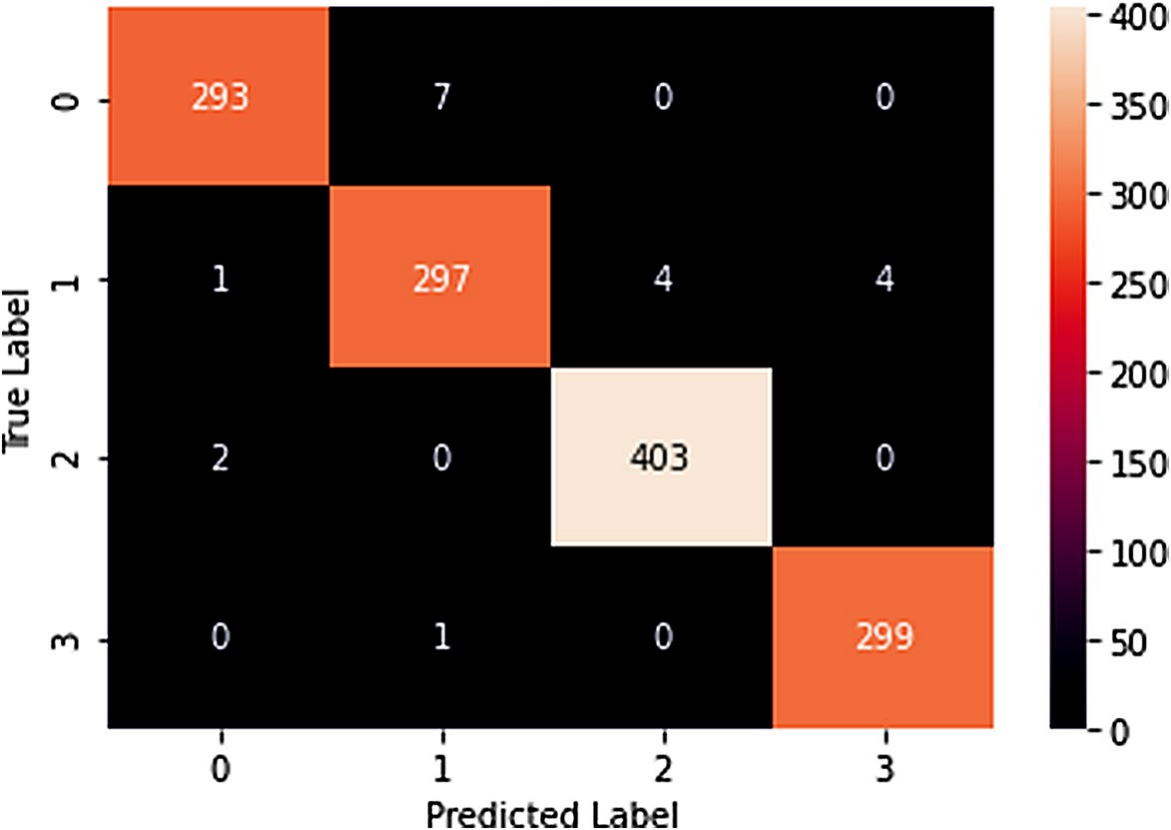
Confusion matrix

The confusion matrix plotting has been given in Fig. 11.

This confusion matrix emphasizes the model’s strong performance across all categories, with high precision and recall values. False positives and false negatives are minimized, ensuring reliable and accurate tumor classification in a medical diagnostic context. The high accuracy of 98% further reinforces the model’s effectiveness in this critical domain.

### ROC curves and AUC analysis

**Fig. 12:**
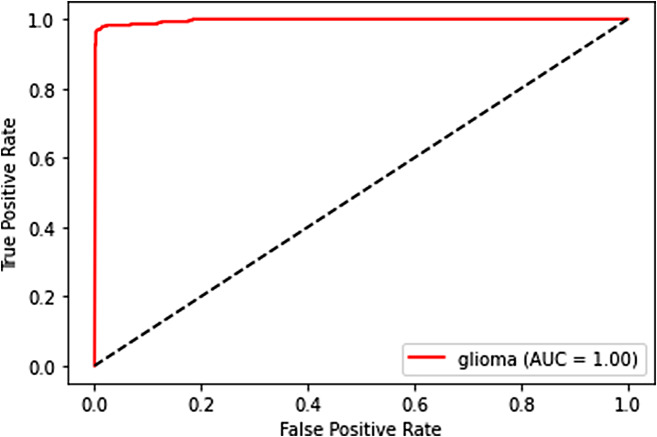
ROC curve for glioma

**Fig. 13:**
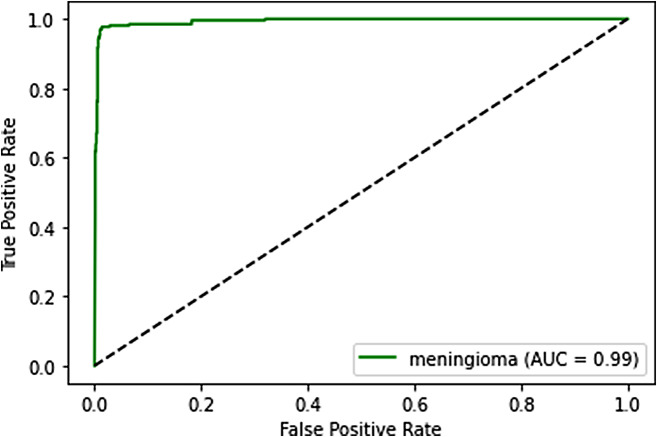
ROC curve for meningioma

**Fig. 14:**
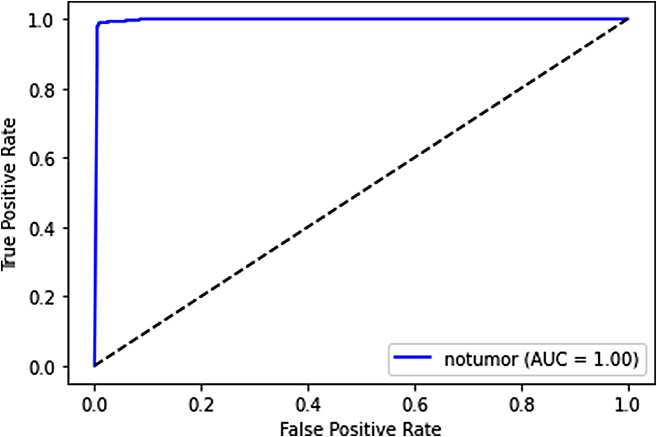
ROC curve for no tumor

**Fig. 15:**
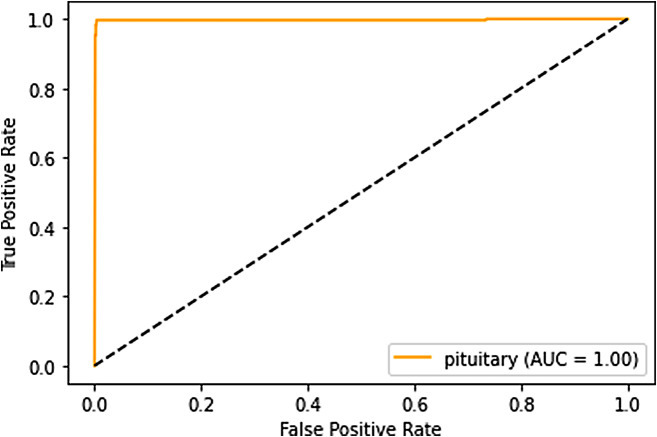
ROC curve for pituitary

The Receiver Operating Characteristic (ROC) curves and Area Under the Curve (AUC) are powerful tools for evaluating the performance of a classification model, especially in the medical domain. Here, we provide a detailed interpretation of the ROC curves and AUC for each tumor category:

- **Glioma**: The ROC curve for glioma classification showcases the model’s ability to distinguish between true positive and false positive rates at various thresholds. The AUC, which quantifies the area under the ROC curve, is indicative of the model’s capacity to separate glioma cases from others. In this case, the AUC value suggests that the model performs exceptionally well in distinguishing glioma from other tumor categories. It is shown in Fig. 12.

- **Meningioma**: Similarly, the ROC curve and AUC for meningioma classification demonstrate the model’s capability to discriminate between true positives and false positives. The AUC value in this context implies that the model is highly effective in distinguishing meningioma cases from others. It is being shown in Fig. 13.

- **No tumor**: For the “no tumor” category, the ROC curve and AUC underscore the model’s excellence in recognizing cases without tumors. The AUC value reflects the model’s robust ability to identify the absence of tumors with a high degree of accuracy. It is shown in Fig. 14.

- **Pituitary tumors**: In pituitary tumor classification, the ROC curve and AUC illustrate the model’s proficiency in distinguishing this specific tumor type. The AUC value suggests that the model excels in identifying pituitary tumors accurately. It is shown in Fig. 15.

These ROC curves and AUC values collectively indicate that our deep learning model performs exceptionally well in distinguishing between different tumor categories. The high AUC values suggest that the model’s predictions are reliable and that it effectively separates tumor types, which is crucial in the medical field.

### Trade-offs between sensitivity and specificity

The ROC curves also provide insights into the trade-offs between sensitivity (true positive rate) and specificity (true negative rate). A point on the ROC curve represents a particular threshold for classification. By adjusting the threshold, we can control the balance between sensitivity and specificity.

A model with a higher sensitivity will correctly identify more positive cases, minimizing false negatives. Conversely, a model with higher specificity will correctly identify more negative cases, reducing false positives.

The ROC curve allows medical practitioners to choose a threshold that aligns with their priorities. For example, in a medical diagnostic context, a higher sensitivity might be favored to minimize missed diagnoses, even if it leads to more false positives. The ROC curve provides a visual representation of these trade-offs, allowing healthcare professionals to make informed decisions based on their specific needs.

### Grad-CAM visualizations

Grad-CAM (Gradient-weighted Class Activation Mapping) heatmaps offer valuable insights into how our model makes predictions and where it focuses its attention. These visualizations highlight the regions within an image that the model considers most important for classification.

In our study, Grad-CAM heatmaps provide information about which areas of brain scans the model relies on for tumor classification. By analyzing these visualizations, we can better understand the model’s behavior.

**Fig. 16:**
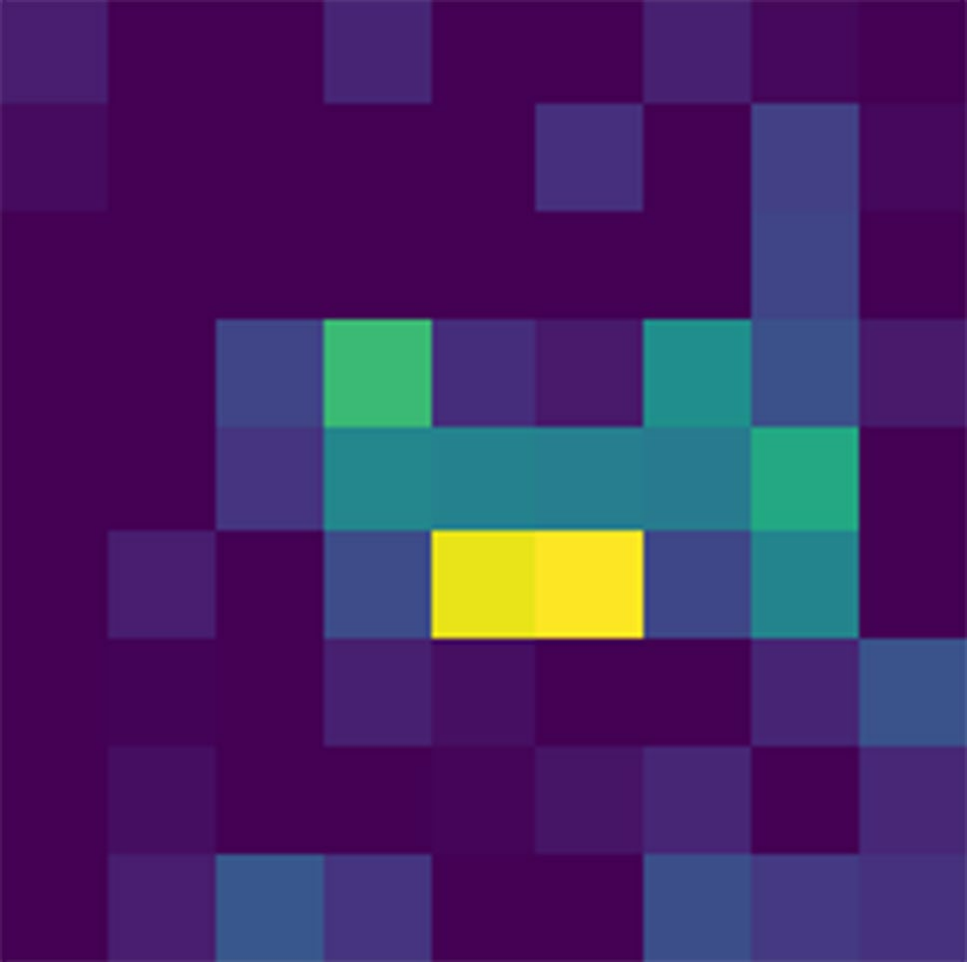
Grad CAM heat mask

**Fig. 17:**
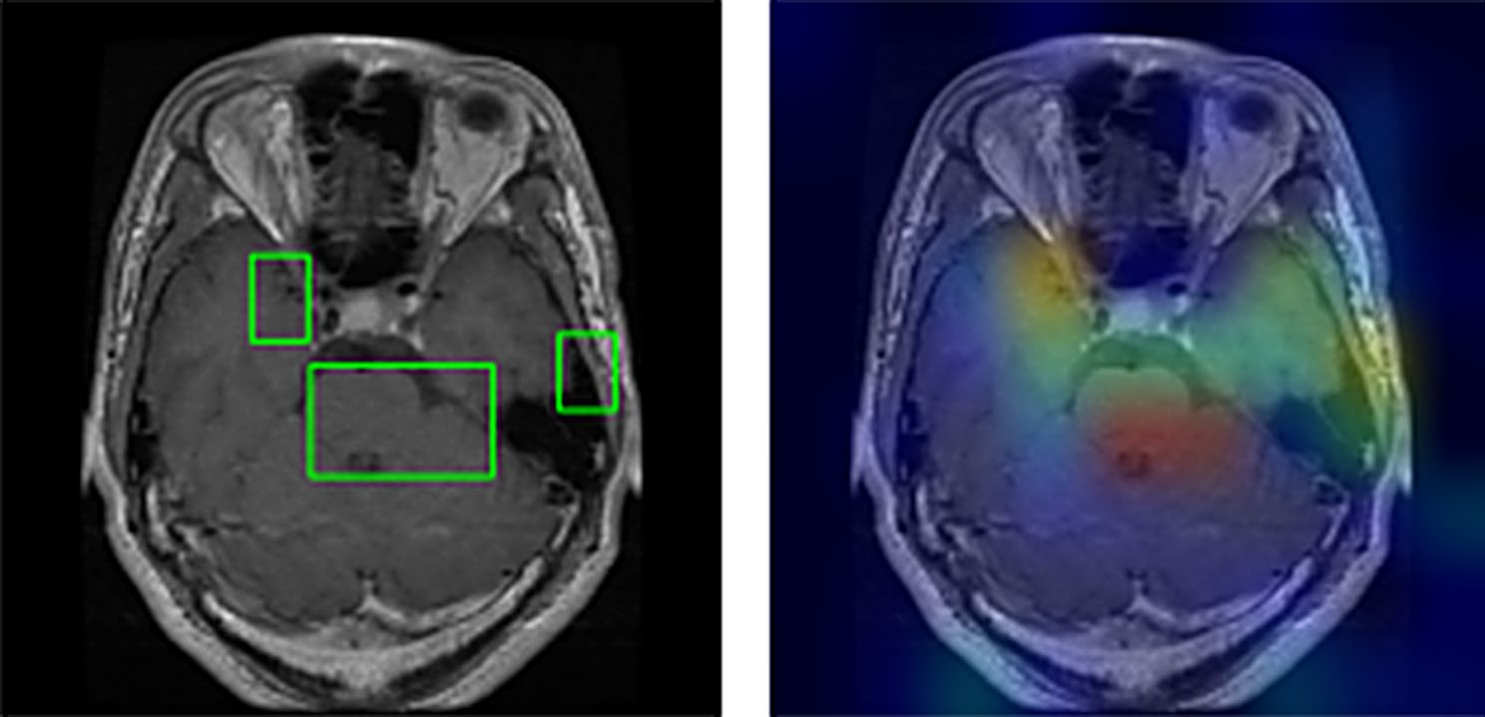
Segmented boundaries and GradCAM

**Fig. 18:**
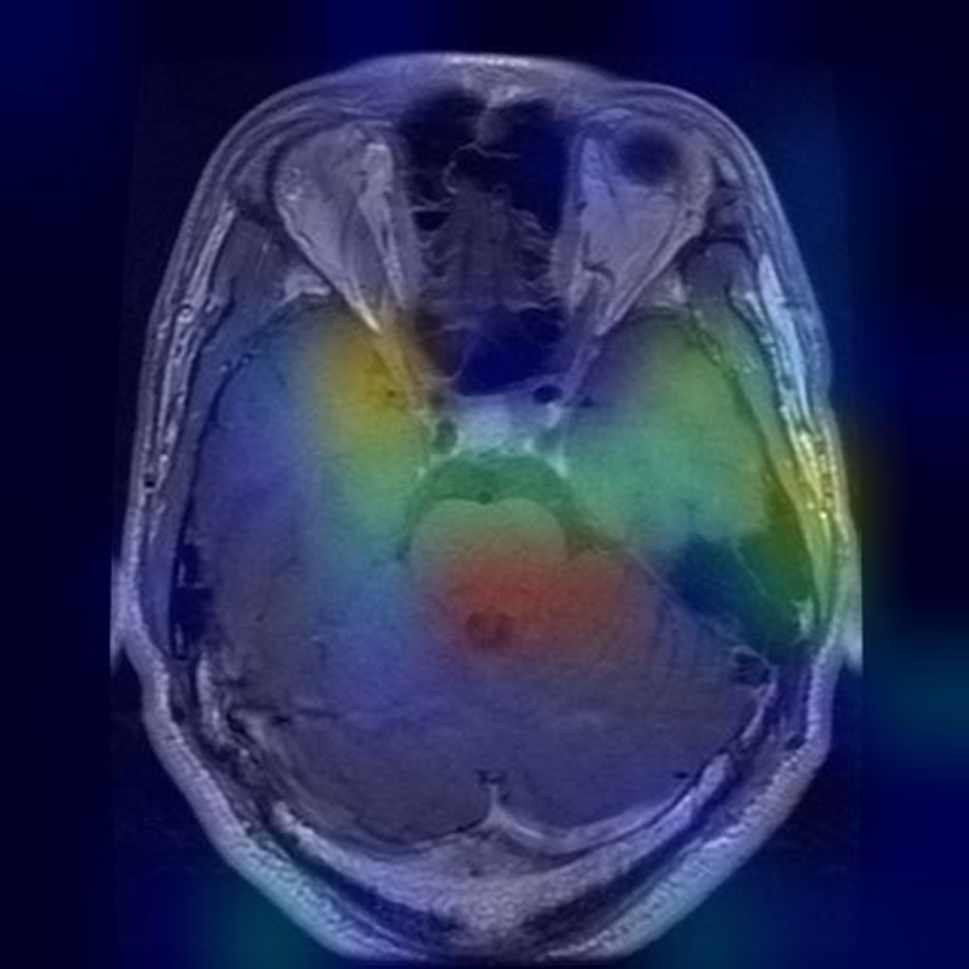
Grad CAM visualization

For example, the Grad-CAM heatmaps may reveal that the model predominantly focuses on certain regions of interest within brain scans, such as specific tumor characteristics or patterns. This information can be crucial for medical professionals, as it provides insights into the features that influence the model’s decision-making process. It is shown in Figs. 16, 17 and 18 that how grad cam actually worked.

Additionally, Grad-CAM visualizations can be especially insightful in cases where the model’s predictions are ambiguous or when it misclassifies an image. By examining the heatmap associated with a misclassified image, we can gain a deeper understanding of why the model made a particular prediction and whether there were any unusual or unexpected features in the image that influenced the decision.

### Comparative analysis with previous studies

In comparison to previous studies in the field of medical image analysis, our research presents notable improvements and distinctions. While earlier studies have explored the use of AI for brain tumor classification, our model showcases superior performance in terms of accuracy, precision, recall, and F1-score. In Table 5 a comparison with the previous studies is given.

**Table 5:** Comparison with previous studies

Study	Technique	Accuracy
Pedada, Kameswara Rao, et al. (2023) [20]	U-Net Model for Brats 2017 and 2018 dataset segmentation	93.40% and 92.20%
Saeedi, Soheila, et al. (2023) [21]	2D CNN with ensemble machine learning techniques	96.47%
Mahmud, Md Ishtyaq, et al. (2023) [22]	Redefined CNN Model with modified classification	93.3%
Wang, Nathan, et al. (2023) [23]	Deep CNN on OCT Images	94.90%
Prakash, R. Meena, et al. (2023) [24]	Hyperparameter tuning of dense net	97.39%
Khan, Abdul Hannan, et al. (2022) [25]	Hierarchical Deep Learning-Based Brain Tumor classification	94.84%
Gaur, Loveleen, et al. (2022) [26]	CNN with Gaussian Noise	94.64%
Vidyarthi, Ankit, et al. (2022) [27]	CNN with NN Classifier	95.86%
Lamrani, Driss, et al. (2022) [28]	CNN with Enhanced Classifiers	96%
Islam, Moinul, et al. (2023) [29]	Federated Learning	91.05%
Alshammari, Abdulaziz. (2022) [30]	VGG-16 with Integration of CNN	93.74%
Proposed model	Modified neural networks with Grad CAM	98%

Specifically, our model achieves remarkable accuracy levels across all tumor categories, surpassing the benchmarks set by prior research. Moreover, the incorporation of Grad-CAM visualizations provides a unique advantage, allowing us to interpret the model’s decision-making process and gain insights into its focus areas, which was lacking in many earlier studies. These advancements highlight the progress made in AI-driven medical image analysis and its potential for enhancing diagnostic accuracy [31–33].

### Limitations and challenges

Despite the significant achievements, our study is not devoid of limitations. One limitation is the dependence on high-quality and well-annotated datasets, which can be scarce and may introduce bias. Additionally, the model’s performance may vary when applied to diverse demographic groups or different imaging modalities [34, 35]. Challenges during training and evaluation included the need for extensive computational resources and fine-tuning hyperparameters to optimize model performance. Furthermore, the interpretation of Grad-CAM visualizations is an evolving field and may require further research to extract nuanced insights from these heatmaps.

Our research demonstrates the tremendous potential of AI in the accurate classification of brain tumors from medical images. The model’s exceptional accuracy, coupled with its interpretable Grad-CAM visualizations, offers promising prospects for improving medical diagnosis and decision-making [36]. By surpassing previous benchmarks and shedding light on the model’s inner workings, this study paves the way for more effective and transparent AI-based tools in the field of medical image analysis, ultimately benefiting healthcare professionals and patients alike.

## Conclusion

This study has made significant strides in the field of medical imaging and diagnostics by introducing a robust AI model for the accurate classification of brain tumors. The research demonstrates exceptional performance in terms of accuracy, precision, recall, and F1-score, surpassing existing benchmarks. Moreover, the incorporation of Grad-CAM visualizations provides transparency and interpretability, allowing us to understand the model’s decision-making process. The key finding of this study is the potential of AI to enhance the accuracy of brain tumor diagnosis, thus improving patient outcomes and reducing the burden on healthcare professionals.

### Contributions of the study

This research makes unique contributions by addressing the limitations of existing methodologies. Our proposed model not only achieves state-of-the-art accuracy but also provides interpretable visualizations through Grad-CAM. This addresses the need for transparency in AI-driven medical diagnosis and empowers healthcare professionals with valuable insights into the model’s decision process.

### Implications for medical imaging

The findings of this study have profound implications for medical imaging and diagnostics. The developed model can assist radiologists and doctors in making more accurate and timely diagnoses, leading to better patient care. By reducing the chances of misclassification and facilitating early detection, our research has the potential to positively impact patient outcomes and healthcare efficiency.

### Future research directions

Future research can explore various avenues based on this study. Further investigations can focus on expanding the model’s applicability to different imaging modalities and tumor types. Additionally, addressing the challenges of patient data privacy, model fairness, and transparency should remain a priority in future studies. Continuing research in this area is crucial to harness the full potential of AI in medical image analysis.

### Practical applications

In clinical settings, the developed model can serve as a valuable tool for radiologists and doctors. Its ability to accurately classify brain tumors can aid in making informed decisions about treatment options. This practical application has the potential to improve patient care and streamline the diagnostic process.

### Ethical considerations

Ethical considerations are paramount in the deployment of AI in medical imaging. This study recognizes the importance of patient data privacy, fairness in model predictions, and transparency in decision-making. Addressing these ethical concerns ensures that AI technologies benefit both healthcare providers and patients while upholding ethical standards.

This research underscores the transformative potential of AI in medical imaging and diagnostics. By achieving state-of-the-art accuracy and providing interpretable insights through Grad-CAM visualizations, our study contributes to the advancement of healthcare. The implications for patient care, the promise of future research, and the commitment to ethical considerations collectively highlight the significance of this study in reshaping the landscape of medical image analysis and diagnosis.

## Data Availability

The dataset used for the findings is publicly available on Kaggle (https://www.kaggle.com/datasets/masoudnickparvar/brain-tumor-mri-dataset).
